# Prevalence of Cardiometabolic Diseases Among Racial and Ethnic Subgroups in Adults — Behavioral Risk Factor Surveillance System, United States, 2013–2021

**DOI:** 10.15585/mmwr.mm7303a1

**Published:** 2024-01-25

**Authors:** Alain K. Koyama, Kai McKeever Bullard, Fang Xu, Stephen Onufrak, Sandra L. Jackson, Ryan Saelee, Yoshihisa Miyamoto, Meda E. Pavkov

**Affiliations:** ^1^Division of Diabetes Translation, National Center for Chronic Disease Prevention and Health Promotion, CDC; ^2^Division for Heart Disease and Stroke Prevention, National Center for Chronic Disease Prevention and Health Promotion, CDC.

**Summary**

**What is already known about this topic?**

Diabetes and cardiovascular disease account for substantial disease prevalence among U.S. adults.

**What is added by this report?**

In this survey of nearly 4 million U.S. adults, prevalence of diagnosed cardiometabolic diseases varied up to twofold among disaggregated racial and ethnic subgroups. Diabetes prevalence among U.S. Asian subgroups ranged from 6.3% among Vietnamese adults to 15.2% among Filipino adults. Among U.S. Hispanic or Latino subgroups, prevalence of angina or coronary heart disease ranged from 3.1% among Cuban adults to 6.3% among Puerto Rican adults.

**What are the implications for public health practice?**

Disaggregated categories of race and ethnicity are crucial in accurately identifying and addressing disparities in cardiometabolic diseases and can be used to help guide prevention programs and development of culturally relevant interventions among U.S. adults.

## Abstract

Although diabetes and cardiovascular disease account for substantial disease prevalence among adults in the United States, their prevalence among racial and ethnic subgroups is inadequately characterized. To fill this gap, CDC described the prevalence of diagnosed cardiometabolic diseases among U.S. adults, by disaggregated racial and ethnic subgroups, among 3,970,904 respondents to the Behavioral Risk Factor Surveillance System during 2013–2021. Prevalence of each disease (diabetes, myocardial infarction, angina or coronary heart disease, and stroke), stratified by race and ethnicity, was based on self-reported diagnosis by a health care professional, adjusting for age, sex, and survey year. Overall, mean respondent age was 47.5 years, and 51.4% of respondents were women. Prevalence of cardiometabolic diseases among disaggregated race and ethnicity subgroups varied considerably. For example, diabetes prevalence within the aggregated non-Hispanic Asian category (11.5%) ranged from 6.3% in the Vietnamese subgroup to 15.2% in the Filipino subgroup. Prevalence of angina or coronary heart disease for the aggregated Hispanic or Latino category (3.8%) ranged from 3.1% in the Cuban subgroup to 6.3% in the Puerto Rican subgroup. Disaggregation of cardiometabolic disease prevalence data by race and ethnicity identified health disparities among subgroups that can be used to better help guide prevention programs and develop culturally relevant interventions.

## Introduction

Cardiometabolic diseases affect a substantial proportion of adults in the United States, including approximately 11% who have diagnosed diabetes,* and 10% who have diagnosed cardiovascular disease (coronary heart disease, heart failure, or stroke) (*1*). Few recent studies have provided estimates of the prevalence of cardiometabolic diseases in disaggregated racial and ethnic subgroups in large nationwide samples (*2*,*3*). Documentation of racial and ethnic disparities in cardiometabolic diseases is typically aggregated because sample sizes are insufficient or because racial and ethnic subgroup data were not collected. These limitations can obscure differences in disease prevalence among disaggregated subgroups that might result from differences in social determinants of health and other drivers of health inequities.

Although racial and ethnic disparities in cardiometabolic disease prevalence have been documented,^†^^,^^§^^,^^¶^ a disaggregated analysis of racial and ethnic groups might better characterize unique patterns of disease prevalence that can more effectively guide prevention and treatment strategies in disaggregated racial and ethnic subgroups at higher risk. To address this gap, CDC evaluated the prevalence of cardiometabolic diseases among selected racial and ethnic subgroups among approximately 4 million adult respondents to the Behavioral Risk Factor Surveillance System (BRFSS) during 2013–2021.

## Methods

### Study Population

BRFSS is an annual random-digit–dialed landline and cellular telephone-based survey representative of noninstitutionalized adults aged ≥18 years from all 50 states, the District of Columbia, and three U.S. territories.** BRFSS includes questions on health-related behavioral risk factors, health care access, and chronic conditions. The study period included 2013, the first year that BRFSS collected disaggregated data on selected race and ethnicity subgroups, through 2021. Among the 4,030,567 total respondents, the analysis excluded 58,743 (1.5%) who were missing data on age, sex, or race and ethnicity, and 1,525 (0.4%) who did not include data on any cardiometabolic diseases. The analysis included the remaining 3,970,904 (98.5%) respondents. This activity was reviewed by CDC, deemed not research, and was conducted consistent with applicable federal law and CDC policy.^††^

### Measurements

Demographic information included age and sex. Respondents who reported Hispanic or Latino (Hispanic) ethnicity were categorized as Hispanic regardless of race. Non-Hispanic respondents were categorized by race. Race and ethnicity choices and corresponding disaggregated subgroups on the questionnaire included Hispanic (Cuban, Mexican, Puerto Rican, or Other Hispanic), non-Hispanic American Indian or Alaska Native (AI/AN), non-Hispanic Asian (Asian [Chinese, Filipino, Indian, Japanese, Korean, Vietnamese, or Other Asian]), non-Hispanic Black or African American (Black), non-Hispanic Pacific Islander (Pacific Islander [Guamanian or Chamorro, Native Hawaiian, Samoan, or other Pacific Islander]), non-Hispanic White (White), non-Hispanic Multiracial, and non-Hispanic Other. Other variables included weight status (underweight, normal, overweight, or obesity as determined by body mass index [BMI] in kg/m2 using World Health Organization criteria for Asian and non-Asian populations) (*4*), physical activity (defined as leisure-time physical activity at least one time in the last month), and smoking status (current, former, or never). Prevalence of cardiometabolic diseases (diabetes [excluding gestational diabetes], myocardial infarction [MI], angina or coronary heart disease [CHD], or stroke) was based on self-reported diagnosis by a physician or other health care professional.

### Statistical Analysis

Prevalence of each cardiometabolic disease was estimated from multivariable logistic models adjusted for age, sex, and survey year. Sample weights and design variables were used to account for the complex survey design. Two-sided p-values <0.05 were considered statistically significant. Analyses were conducted using SAS (version 9.4; SAS Institute) and SUDAAN (version 11.0.1; Research Triangle Institute).

## Results

Among a total of 3,970,904 adults, the mean respondent age was 47.5 years, and 51.4% were women (Table 1). Among both aggregated groups and disaggregated subgroups, the proportion of adults reporting less than a high school education varied from 2.7% among Korean adults to 41.5% among Mexican adults. The proportion of adults with obesity ranged from 12.0% among Chinese adults to 51.2% among Samoan adults.

**TABLE 1:** Characteristics of adults aged ≥18 years, by race and ethnicity — Behavioral Risk Factor Surveillance System, United States, 2013–2021

Group (no of persons; %)*	Mean age, yrs (95% CI)	% (95% CI)							
		Women	Less than high school education	Weight status^†^		Physical activity in past month^¶^	Smoking status^§^		
				Overweight	Obese		Never	Former	Current
**Total (3,970,904)**	**47.5 (47.5–47.6)**	**51.4 (51.3–51.5)**	**13.6 (13.5–13.7)**	**35.7 (35.6–35.8)**	**30.8 (30.7–30.9)**	**74.9 (74.8–75.0)**	**59.8 (59.7–60.0)**	**24.2 (24.1–24.3)**	**15.9 (15.9–16.0)**
**American Indian or Alaska Native, NH (66,022; 1.0%)**	**46.3 (46.0–46.6)**	**49.8 (48.9–50.6)**	**20.3 (19.6–21.1)**	**33.6 (32.8–34.4)**	**37.1 (36.2–37.9)**	**71.6 (70.8–72.4)**	**46.3 (45.5–47.2)**	**24.7 (24.0–25.5)**	**28.9 (28.1–29.7)**
**Asian, NH (90,298; 5.3%)**	**40.9 (40.7–41.2)**	**50.1 (49.4–50.8)**	**4.7 (4.4–5.1)**	**41.5 (40.7–42.2)**	**20.5 (19.9–21.0)**	**79.9 (79.4–80.5)**	**80.2 (79.6–80.8)**	**12.2 (11.7–12.6)**	**7.6 (7.3–8.0)**
Chinese (14,035; 1.0%)	39.5 (38.9–40.0)	52.6 (51.0–54.1)	3.1 (2.5–3.9)	38.1 (36.5–39.8)	12.0 (10.9–13.2)	81.6 (80.2–82.8)	85.3 (84.1–86.5)	8.6 (7.7–9.6)	6.1 (5.4–6.9)
Filipino (17,063; 0.7%)	45.7 (44.9–46.5)	59.9 (57.9–61.8)	6.1 (5.1–7.2)	43.0 (41.0–45.2)	27.4 (25.6–29.3)	77.8 (76.1–79.5)	75.2 (73.4–77.0)	15.7 (14.3–17.2)	9.0 (7.9–10.3)
Indian (17,851; 1.2%)	40.4 (40.0–40.8)	43.0 (41.7–44.4)	3.7 (3.2–4.3)	46.6 (45.2–48.0)	22.5 (21.3–23.7)	80.4 (79.3–81.5)	84.9 (84.0–85.8)	9.5 (8.8–10.2)	5.6 (5.1–6.2)
Japanese (12,663; 0.3%)	55.4 (54.4–56.4)	57.6 (54.8–60.4)	3.8 (2.7–5.3)	36.8 (34.0–39.6)	25.8 (23.1–28.7)	80.7 (78.3–82.9)	61.9 (58.9–64.8)	28.7 (26.0–31.5)	9.4 (7.7–11.5)
Korean (4,355; 0.3%)	37.4 (36.4–38.3)	50.6 (47.6–53.6)	2.7 (2.0–3.7)	40.1 (37.0–43.3)	16.1 (14.0–18.4)	81.6 (78.9–84.0)	68.5 (65.7–71.1)	18.0 (15.9–20.4)	13.5 (11.7–15.5)
Vietnamese (3,142; 0.3%)	36.8 (35.7–37.8)	46.9 (43.5–50.2)	7.0 (4.8–10.2)	37.6 (34.4–40.9)	13.5 (11.6–15.6)	78.3 (75.6–80.7)	81.3 (78.7–83.6)	9.7 (8.0–11.8)	9.0 (7.4–10.9)
Other Asian (21,189; 1.5%)	39.1 (38.6–39.6)	49.0 (47.6–50.4)	6.0 (5.3–6.8)	40.8 (39.4–42.2)	22.5 (21.3–23.7)	79.2 (78.1–80.3)	80.4 (79.3–81.5)	11.5 (10.6–12.4)	8.1 (7.4–8.7)
**Black or African American, NH (315,725; 11.8%)**	**45.7 (45.6–45.8)**	**54.2 (53.8–54.5)**	**14.4 (14.1–14.6)**	**33.5 (33.2–33.8)**	**39.6 (39.2–39.9)**	**70.1 (69.8–70.4)**	**65.9 (65.5–66.2)**	**16.0 (15.8–16.2)**	**18.1 (17.9–18.4)**
**Pacific Islander, NH (16,421; 0.2%)**	**40.6 (40.1–41.2)**	**49.1 (47.3–50.8)**	**11.8 (10.7–13.0)**	**32.6 (30.9–34.4)**	**35.5 (33.8–37.3)**	**76.7 (75.2–78.2)**	**62.3 (60.5–64.0)**	**18.8 (17.4–20.2)**	**19.0 (17.6–20.4)**
Guamanian or Chamorro (5,163; 0.02%)	42.0 (40.7–43.3)	50.6 (46.6–54.6)	18.9 (16.3–21.9)	36.8 (32.4–41.4)	40.6 (36.8–44.5)	72.5 (69.1–75.6)	53.6 (49.7–57.5)	18.1 (15.6–20.9)	28.3 (25.0–31.7)
Native Hawaiian (3,411; 0.04%)	43.6 (42.4–44.8)	51.1 (47.6–54.6)	8.3 (6.6–10.3)	32.7 (29.5–36.1)	38.1 (34.9–41.5)	79.3 (76.3–82.1)	55.2 (51.7–58.8)	24.7 (21.8–27.8)	20.1 (17.3–23.2)
Samoan (938; 0.02%)	37.6 (36.2–39.0)	42.8 (36.9–49.0)	10.5 (7.5–14.4)	29.2 (23.4–35.7)	51.2 (44.5–57.8)	75.5 (70.0–80.3)	56.5 (49.9–62.8)	17.7 (12.6–24.3)	25.8 (21.0–31.3)
Other Pacific Islander (6,909; 0.1%)	40.0 (39.3–40.8)	49.1 (46.6–51.5)	11.6 (10.0–13.3)	32.3 (30.0–34.7)	31.7 (29.4–34.1)	77.0 (74.8–79.0)	66.6 (64.1–68.9)	17.5 (15.7–19.4)	16.0 (14.2–17.9)
**White, NH (3,041,848; 62.8%)**	**50.3 (50.2–50.3)**	**51.4 (51.3–51.5)**	**8.3 (8.2–8.4)**	**35.3 (35.2–35.5)**	**29.3 (29.2–29.4)**	**77.0 (76.9–77.1)**	**54.6 (54.5–54.7)**	**28.6 (28.5–28.7)**	**16.7 (16.7–16.8)**
**Hispanic or Latino (333,673; 17.0%)**	**41.3 (41.2–41.4)**	**50.3 (50.0–50.7)**	**35.5 (35.2–35.8)**	**37.3 (36.9–37.6)**	**33.6 (33.3–34.0)**	**68.4 (68.1–68.7)**	**70.7 (70.3–71.0)**	**16.9 (16.6–17.1)**	**12.5 (12.2–12.7)**
Cuban (7,566; 0.5%)	47.9 (47.0–48.8)	49.2 (46.9–51.5)	18.9 (17.0–21.0)	37.8 (35.4–40.2)	30.7 (28.5–33.1)	65.6 (63.2–67.9)	62.6 (60.2–64.9)	20.0 (18.1–21.9)	17.4 (15.6–19.5)
Mexican (149,206; 9.2%)	40.4 (40.2–40.5)	49.8 (49.3–50.3)	41.5 (41.0–42.0)	37.5 (37.0–38.1)	36.2 (35.7–36.7)	69.6 (69.1–70.1)	71.5 (71.1–72.0)	16.3 (16.0–16.7)	12.2 (11.8–12.5)
Puerto Rican (75,871; 2.4%)	44.8 (44.6–45.0)	52.9 (52.3–53.5)	22.9 (22.4–23.5)	35.5 (34.9–36.2)	33.1 (32.5–33.7)	61.1 (60.5–61.7)	65.4 (64.8–66.0)	19.0 (18.5–19.5)	15.6 (15.1–16.1)
Other Hispanic or Latino (101,030; 4.9%)	40.7 (40.5–40.9)	50.2 (49.6–50.8)	32.4 (31.8–33.0)	37.6 (37.0–38.2)	29.4 (28.8–30.0)	70.2 (69.6–70.7)	72.7 (72.1–73.2)	16.5 (16.1–17.0)	10.8 (10.4–11.2)
**Multiracial, NH (80,117; 1.4%)**	**42.3 (42.0–42.6)**	**51.3 (50.6–52.1)**	**11.2 (10.7–11.8)**	**32.2 (31.5–32.9)**	**31.8 (31.1–32.5)**	**78.2 (77.6–78.8)**	**53.6 (52.8–54.3)**	**23.2 (22.5–23.8)**	**23.3 (22.6–23.9)**
**Other, NH (26,800; 0.5%)**	**47.8 (47.4–48.3)**	**46.3 (45.1–47.5)**	**12.7 (11.8–13.6)**	**35.8 (34.6–37.0)**	**27.5 (26.3–28.6)**	**75.0 (74.0–76.0)**	**59.2 (58.0–60.4)**	**23.0 (22.0–24.1)**	**17.8 (16.8–18.7)**

**Abbreviation**: NH = non-Hispanic.

* Row percentages are weighted and represent the proportion of the total population for each group or disaggregated subgroup.

^†^ Categories are based on World Health Organization thresholds of body mass index (kg/m^2^) (non-Asian: <18.5 [underweight], 18.5–24.9 [normal], 25.0–29.9 [overweight], ≥30 [obese]; Asian: <18.5 [underweight], 18.5–22.9 [normal], 23.0–27.4 [overweight], or ≥27.5 [obese]).

^§^ Current smokers are defined as respondents who reported having smoked at least 100 cigarettes in their lifetime and now smoke every day or some days. Former smokers are those who reported having smoked at least 100 cigarettes in their lifetime and currently do not smoke. Never smokers are those who reported they had not smoked at least 100 cigarettes in their lifetime.

^¶^ Defined as self-reported leisure-time physical activity at least once in the past month.

### Diabetes

The overall prevalence of diabetes was 10.9% (Table 2); the range among aggregated race and ethnicity groups was from 9.1% among White adults to 16%–17% among AI/AN, Black, and Pacific Islander adults. Prevalence was 11.5% for the aggregated Asian category; among disaggregated Asian subgroups, prevalence ranged from 6.3% for Vietnamese adults to 15.2% for Filipino adults. Diabetes prevalence among all subgroups was highest among Samoan adults (20.3%).

**TABLE 2:** Adjusted prevalence of cardiometabolic diseases among adults aged ≥18 years, by race and ethnicity — Behavioral Risk Factor Surveillance System, United States, 2013–2021

Group (no. of persons)	% (95% CI)*			
	Diabetes	Angina or coronary heart disease	Myocardial infarction	Stroke
**Total (3,970,904)**	**10.9 (10.8–11.0) **	**4.1 (4.1–4.2) **	**4.3 (4.3–4.3) **	**3.2 (3.1–3.2) **
**American Indian or Alaska Native, NH (66,022)**	**16.8 (16.2–17.4) **	**6.1 (5.7–6.4) **	**7.9 (7.5–8.3)**	**6.2 (5.9–6.6) **
**Asian, NH (90,298)**	**11.5 (11.0–12.0) **	**2.8 (2.5–3.1) **	**2.6 (2.4–2.9) **	**1.8 (1.6–2.1) **
Chinese (14,035)	7.0 (6.1–8.1)	1.7 (1.3–2.4)	1.7 (1.3–2.3)	1.3 (1.0–1.8)
Filipino (17,063)	15.2 (13.8–16.8)	3.6 (2.9–4.6)	3.1 (2.3–4.1)	2.4 (1.8–3.2)
Indian (17,851)	14.7 (13.7–15.7)	3.5 (2.9–4.1)	3.3 (2.8–4.0)	1.4 (1.1–1.8)
Japanese (12,663)	9.6 (8.1–11.4)	2.2 (1.6–3.1)	1.9 (1.3–2.8)	2.1 (1.5–2.9)
Korean (4,355)	7.2 (5.8–9.0)	1.1 (0.7–1.8)	1.7 (1.1–2.5)	3.0 (1.5–5.8)^†^
Vietnamese (3,142)	6.3 (4.7–8.4)	1.7 (0.9–3.1)	2.3 (1.3–3.9)	2.0 (1.1–3.5)
Other Asian (21,189)	11.8 (10.8–13.0)	3.0 (2.4–3.6)	2.9 (2.4–3.4)	1.8 (1.4–2.3)
**Black or African American, NH (315,725)**	**16.2 (16.0–16.4) **	**4.1 (3.9–4.2) **	**4.6 (4.4–4.7) **	**5.0 (4.9–5.1) **
**Pacific Islander, NH (16,421)**	**16.6 (15.2–18.0) **	**5.1 (4.1–6.3) **	**5.4 (4.5–6.4) **	**4.3 (3.6–5.3) **
Guamanian or Chamorro (5,163)	19.9 (17.1–23.1)	7.2 (4.8–10.7)	6.1 (4.7–8.0)	5.8 (3.9–8.6)
Native Hawaiian (3,411)	14.6 (12.1–17.4)	5.6 (3.7–8.4)	7.6 (5.5–10.4)	6.1 (4.2–8.9)
Samoan (938)	20.3 (14.8–27.1)	5.1 (2.8–9.2)^†^	5.6 (3.5–8.8)	4.3 (2.6–7.1)
Other Pacific Islander (6,909)	16.0 (14.2–18.0)	4.5 (3.2–6.2)	4.5 (3.3–6.0)	3.4 (2.5–4.6)
**White, NH (3,041,848)**	**9.1 (9.0–9.1)**	**4.2 (4.2–4.3)**	**4.2 (4.2–4.3) **	**2.9 (2.9–3.0) **
**Hispanic or Latino (333,673)**	**15.3 (15.1–15.6) **	**3.8 (3.7–4.0) **	**4.4 (4.3–4.6) **	**2.9 (2.8–3.0) **
Cuban (7,566)	11.0 (9.7–12.4)	3.1 (2.3–4.0)	4.5 (3.6–5.5)	2.4 (1.8–3.1)
Mexican (149,206)	16.7 (16.3–17.1)	3.2 (2.9–3.4)	4.1 (3.9–4.3)	2.8 (2.6–3.0)
Puerto Rican (75,871)	16.7 (16.2–17.1)	6.3 (6.0–6.6)	5.3 (5.0–5.6)	3.2 (2.9–3.5)
Other Hispanic or Latino (101,030)	12.6 (12.2–13.1)	3.5 (3.3–3.8)	4.4 (4.2–4.7)	3.0 (2.8–3.2)
**Multiracial, NH (80,117)**	**12.8 (12.3–13.3) **	**5.6 (5.3–6.0) **	**6.5 (6.1–6.9) **	**5.4 (5.1–5.8) **
**Other, NH (26,800)**	**11.3 (10.6–12.0) **	**4.4 (4.0–4.8) **	**4.6 (4.2–5.0) **	**3.9 (3.5–4.4) **

**Abbreviation:** NH = non-Hispanic.

* Prevalence is adjusted for age, sex, and survey year. Each cardiometabolic disease (diabetes, myocardial infarction, angina or coronary heart disease, or stroke) is based on self-reported diagnosis by a health care professional.

^†^ Estimate has a relative SE ≥0.30 and is therefore considered statistically unreliable.

### Angina, Coronary Heart Disease, Myocardial Infarction, and Stroke

The overall prevalence of angina or CHD was 4.1%; the range among aggregated race and ethnicity groups was from 2.8% among Asian adults to 6.1% among AI/AN adults. Among subgroups, prevalence ranged from 1.1% among Korean adults to 7.2% among Guamanian or Chamorro adults. The prevalence for the aggregated Hispanic category was 3.8% and ranged from 3.1% among Cuban adults to 6.3% among Puerto Rican adults. Overall prevalence of MI was 4.3%; the pattern of variation among aggregated race and ethnicity groups was similar to that of angina or CHD. Prevalence of stroke was 3.2% overall, ranging from 1.8% among Asian adults to 6.2% among AI/AN adults.

## Discussion

Findings from this study illustrate pronounced differences in cardiometabolic disease prevalence among racial and ethnic subgroups, with the largest variation occurring in diabetes prevalence. Among Hispanic subgroups, diabetes prevalence was highest for Mexican and Puerto Rican adults and lowest for Cuban adults. Among Asian subgroups, diabetes prevalence was highest for Filipino and Indian adults and lowest for Chinese, Korean, and Vietnamese adults.

### Hispanic Subgroups

In the current study, although the prevalence of diabetes was the same among both Puerto Rican and Mexican adults, prevalence of angina or CHD was approximately twice as high among Puerto Rican adults. In a cohort study during 2008–2011, elevated prevalence of cardiometabolic diseases in the Puerto Rican subgroup was associated with a higher prevalence of cardiovascular risk factors such as smoking (*5*). Increased acculturation was also associated with a higher prevalence of cardiovascular disease risk factors (*5*). Prevalence of current smoking was higher in the Puerto Rican group than in the Mexican group, both in the current study and during 2008–2011. The prevalence of physical activity during the past month was lower in the Puerto Rican group in the current study; Puerto Rican adults had higher educational attainment and a slightly lower prevalence of overweight and obesity. It is unclear how this overall combination of risk factors might explain the observed pattern of cardiometabolic diseases in the current study.

### Asian and Pacific Islander Subgroups

A higher prevalence of most cardiometabolic diseases among Filipino and Indian adults compared with other Asian subgroups might be partly attributed to differences in the prevalences of hypertension, hyperlipidemia, and overweight or obesity (*2*,*6*). In the current study, prevalence of overweight or obesity was higher among Filipino and Indian adults than among other Asian subgroups, as has been reported previously (*2*,*6*). Because of small sample sizes, high variability in prevalence estimates makes subgroup disparities difficult to infer among Pacific Islander adults. In the current study, educational attainment and prevalence of physical activity in the past month were higher among Native Hawaiian adults compared with other Pacific Islander subgroups, although variability of prevalence estimates overall was high. Similarly, a previous study of 561 patients hospitalized with ischemic stroke suggested that Native Hawaiian adults might have a more favorable cardiovascular profile than do other Pacific Islander adults (*7*).

### Cardiometabolic Disease and BMI

Evidence suggests that a specific distribution of ectopic fat as a measure of adiposity might be a stronger marker of cardiometabolic diseases than BMI (*8*). The generally low BMI but high diabetes risk among Asian populations (*9*) might provide unique opportunities to better understand diabetes etiology and shed light on the paradoxical continuing increase in obesity rates^§§^ concurrent with recent declining diabetes incidence in the United States.^¶¶^

### Limitations

The findings in this report are subject to at least three limitations. First, self-reported information might be subject to bias, including underreporting of disease prevalence. Underreporting, such as that associated with undiagnosed disease, might affect prevalence estimates to different degrees in different groups. Second, because the survey questionnaire did not collect disaggregated data for AI/AN (e.g., by tribal affiliation or tribal enrollment status), Black, and White adults, potential subgroup disparities could not be assessed among these groups. Finally, information was not available on other relevant factors related to disaggregated race and ethnicity, such as country of birth or English language proficiency.

### Implications for Public Health Practice

Continued collection and analysis of disaggregated data could enable more accurate characterization of racial and ethnic disparities in cardiometabolic diseases among U.S. adults. Collection of clinical data with comparable definitions of race and ethnicity might allow comparison of prevalence estimates with self-reported data. Further, as availability of disaggregated data increases, increased use of such data might allow implementation of better tailored disease prevention and management programs. A greater body of evidence might allow an evaluation of how best to implement and financially sustain these programs, train staff members in cultural competency, and maximize the effectiveness in reducing health disparities.

## Footnotes

*
https://www.cdc.gov/diabetes/data/statistics-report/index.html#anchor_40084

†
https://www.cdc.gov/diabetes/data/statistics-report/appendix.html#tabs-1-1

§
https://www.cdc.gov/heartdisease/facts.htm

¶
https://www.cdc.gov/nchs/data/hus/2018/013.pdf

**
https://www.cdc.gov/brfss/about/index.htm

†† 45 C.F.R. part 46.102(l)(2), 21 C.F.R. part 56; 42 U.S.C. Sect. 241(d); 5 U.S.C. Sect. 552a; 44 U.S.C. Sect. 3501 et seq.

§§
https://www.cdc.gov/nchs/data/hus/2019/026-508.pdf

¶¶
https://www.cdc.gov/diabetes/data/statistics-report/index.html#anchor_63700
